# Pulmonary Epithelioid Trophoblastic Tumor Without Demonstrable Uterine Involvement: A Case Report and Literature Review Highlighting Diagnostic Challenges and Multidisciplinary Management

**DOI:** 10.3390/jcm15197578

**Published:** 2026-09-29

**Authors:** Kai Zhang, Juan Zhang, Xingkui Xie, Jinghong Jiang

**Affiliations:** Department of Gynecology and Obstetrics, Zhongnan Hospital of Wuhan University, 169 Donghu Road, Wuchang District, Wuhan 430071, China; zhangkai530685922@163.com (K.Z.); zn003075@whu.edu.cn (J.Z.); zn004343@whu.edu.cn (X.X.)

**Keywords:** epithelioid trophoblastic tumor, fertility preservation, lung cancer, misdiagnosis

## Abstract

**Background:** Pulmonary epithelioid trophoblastic tumor (ETT) without demonstrable uterine involvement is an exceedingly rare form of gestational trophoblastic neoplasia. Its non-specific presentation often results in misdiagnosis, commonly as ectopic pregnancy. **Case Presentation:** A 39-year-old woman, para 2, presented with abnormal uterine bleeding and an elevated serum β-human chorionic gonadotropin (β-hCG) level of 978 mIU/mL. Despite the absence of sonographic evidence for either intrauterine or ectopic pregnancy, she was initially misdiagnosed with ectopic gestation. Following a failed systemic methotrexate therapy, she underwent a non-diagnostic laparoscopic bilateral salpingectomy and curettage; however, her β-hCG levels continued to rise persistently. A computed tomography (CT) scan performed one month later revealed a solitary mass measuring 57 × 40 mm in the right lower lobe of the lung. A multidisciplinary team subsequently decided on a right lower lobectomy. Postoperatively, the patient’s β-hCG levels normalized rapidly. Histopathological examination of the lung mass confirmed the diagnosis of pulmonary ETT without demonstrable uterine involvement. She received 3 cycles of combination EMA/Co chemotherapy (etoposide, methotrexate, and actinomycin D, followed by cyclophosphamide and vincristine), and the serum β-hCG level dropped to normal. At the 3-year posttreatment follow-up, the patient reported regular menstrual cycles, with serum β-hCG levels consistently within normal limits. **Conclusions:** This case highlights that persistent elevation of β-hCG in the absence of pelvic pathology should prompt a comprehensive investigation for an extrauterine source. Pulmonary ETT must be included in the differential diagnosis for reproductive-aged women presenting with a pulmonary mass and elevated β-hCG levels. A multidisciplinary approach enabled both definitive diagnosis and fertility-sparing management in this patient; broader conclusions about treatment efficacy await larger series.

## 1. Introduction

Gestational trophoblastic disease (GTD) encompasses a spectrum of pregnancy-related disorders ranging from benign hydatidiform moles to malignant gestational trophoblastic neoplasia (GTN). Epithelioid trophoblastic tumor (ETT) is the rarest among the four GTN subtypes, which comprise invasive mole, choriocarcinoma, placental site trophoblastic tumor (PSTT), and ETT, accounting for less than 1–2% of cases [1,2]. First characterized by Shih and Kurman in 1998, ETT arises from chorionic-type intermediate trophoblast cells and is distinguished by its epithelioid morphology, relatively slow growth, and notable resistance to single-agent chemotherapy [3].

Pulmonary ETT, which originates in the lung without evidence of a uterine primary, is exceptionally rare [4]. Our systematic search identified 30 cases of pulmonary ETT without demonstrable uterine involvement published in the English literature, and all are documented in isolated case reports or small case series in Supplemental Table S1. Its clinical presentation overlaps with both ectopic pregnancy (irregular bleeding, elevated β-hCG) and primary lung malignancies (cough, hemoptysis, incidental nodule). The diagnostic challenge is amplified by ETT’s characteristically low β-hCG levels (typically hundreds rather than thousands or tens of thousands of mIU/mL), which often prompt empirical ectopic pregnancy management before the correct diagnosis is considered [5].

Surgical resection is the cornerstone of ETT treatment, given the tumor’s chemoresistance. For metastatic or refractory cases, multi-agent regimens such as EMA/CO (etoposide, methotrexate, actinomycin D, cyclophosphamide, and vincristine) or EMA/EP (etoposide, methotrexate, actinomycin D/etoposide, and cisplatin) are recommended [6]. Both regimens are considered acceptable first-line options for high-risk GTN, with EMA/EP generally reserved for EMA/CO-resistant cases or as an alternative in specific clinical settings. Recent transcriptomic studies have identified phosphatidylinositol 3-kinase (PI3K)-Akt-mammalian target of rapamycin (mTOR) pathway activation and near-universal programmed death-ligand 1 (PD-L1) expression in ETT, providing molecular rationales for targeted and immunotherapeutic approaches in chemoresistant disease [7,8]. A 2026 narrative review of checkpoint inhibition in GTN reported complete remission rates of 70–80% with anti-PD-1/PD-L1 agents in multidrug-resistant patients, though ETT-specific immunotherapy data remain limited to case reports [9,10].

A critical diagnostic gap is the absence of a standardized protocol for investigating unexplained, persistent β-hCG elevation in reproductive-aged women after excluding intrauterine and adnexal pathology. As demonstrated in our case and in recently independently reported cases [11,12], this gap leads to repeated cycles of empirical therapy and diagnostic delays of months to years. The present report has three objectives: (i) present an illustrative case of pulmonary ETT; (ii) provide a systematic synthesis of the clinical presentation, β-hCG dynamics, and outcomes across all available recent reports; and (iii) propose a structured diagnostic algorithm grounded in this synthesis to facilitate earlier recognition and definitive surgical management.

## 2. Case Report

A 39-year-old woman with a history of regular menstrual cycles initially presented with abnormal uterine bleeding for half a month, prompting a visit to a local hospital where she was mistakenly diagnosed with pregnancy due to an elevated serum beta-human chorionic gonadotropin (β-hCG) level of 978 mIU/mL. She had a history of two vaginal deliveries and used an intrauterine device for contraception. The most recent pregnancy was a vaginal delivery of a live baby three years ago. The patient presented with 50 days of amenorrhea followed by approximately two weeks of irregular vaginal bleeding. Transvaginal ultrasonography revealed an endometrial thickness of 10 mm, with no evidence of an intrauterine or adnexal gestational sac. The initial diagnosis was ectopic pregnancy. Despite no positive findings on transvaginal ultrasound, an initial suspicion of ectopic pregnancy led to the administration of methotrexate (MTX) therapy. A 50 mg oral dose of mifepristone was administered in combination with conservative traditional Chinese medicine therapy. However, her β-HCG level showed a slight decrease initially but then rapidly rose to 1100 mIU/mL. Subsequent laparoscopic bilateral salpingectomy and curettage were performed. An intrauterine device, which had been in place for contraception, was removed at the time of curettage. But postoperative pathological examination revealed no villous tissue and showed proliferative endometrium. β-HCG level remained elevated. She remained asymptomatic, reporting no abdominal pain, respiratory symptoms, or chest discomfort. Nonetheless, her menstrual cycles did not resume.

**Figure 1:**
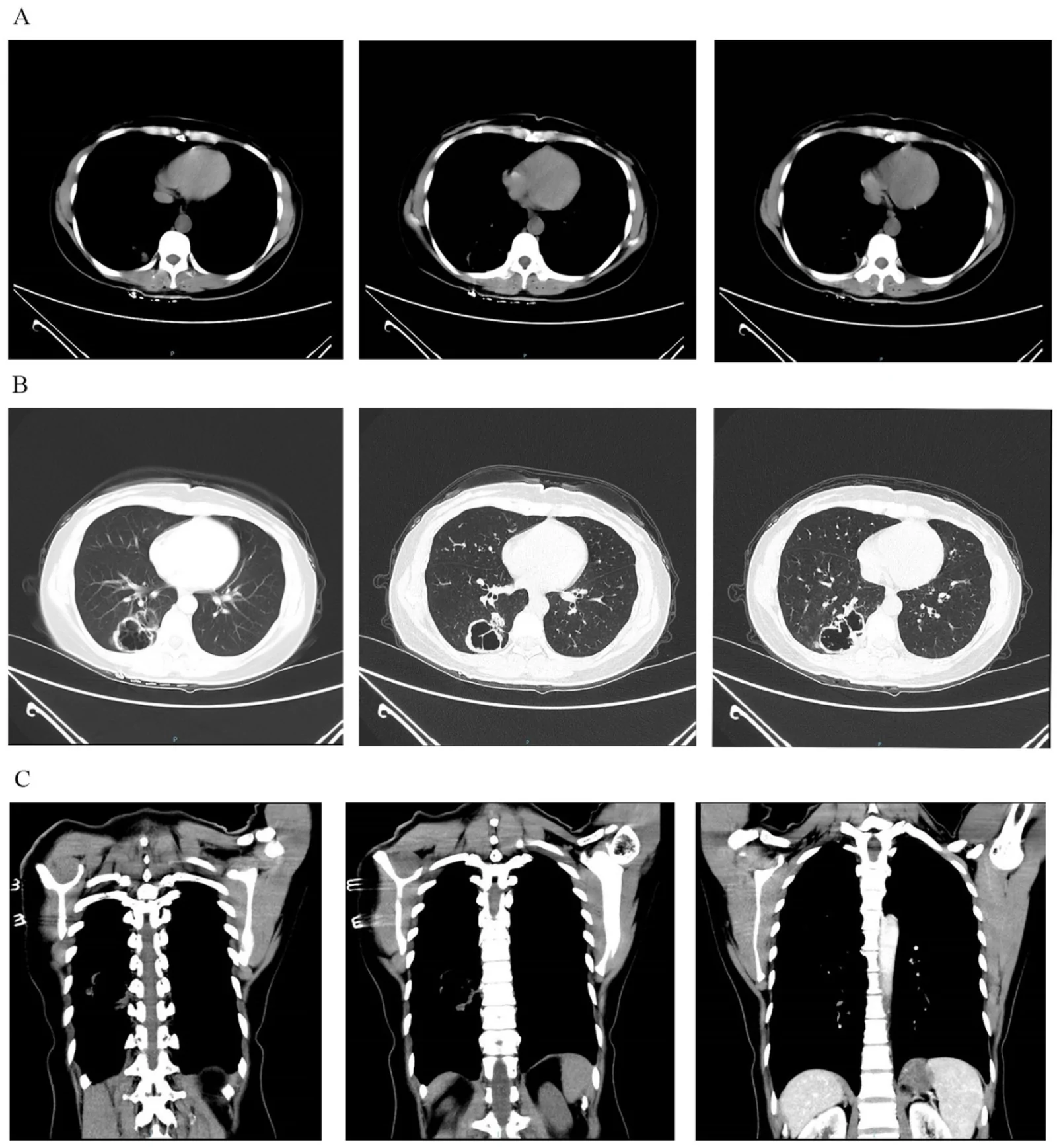

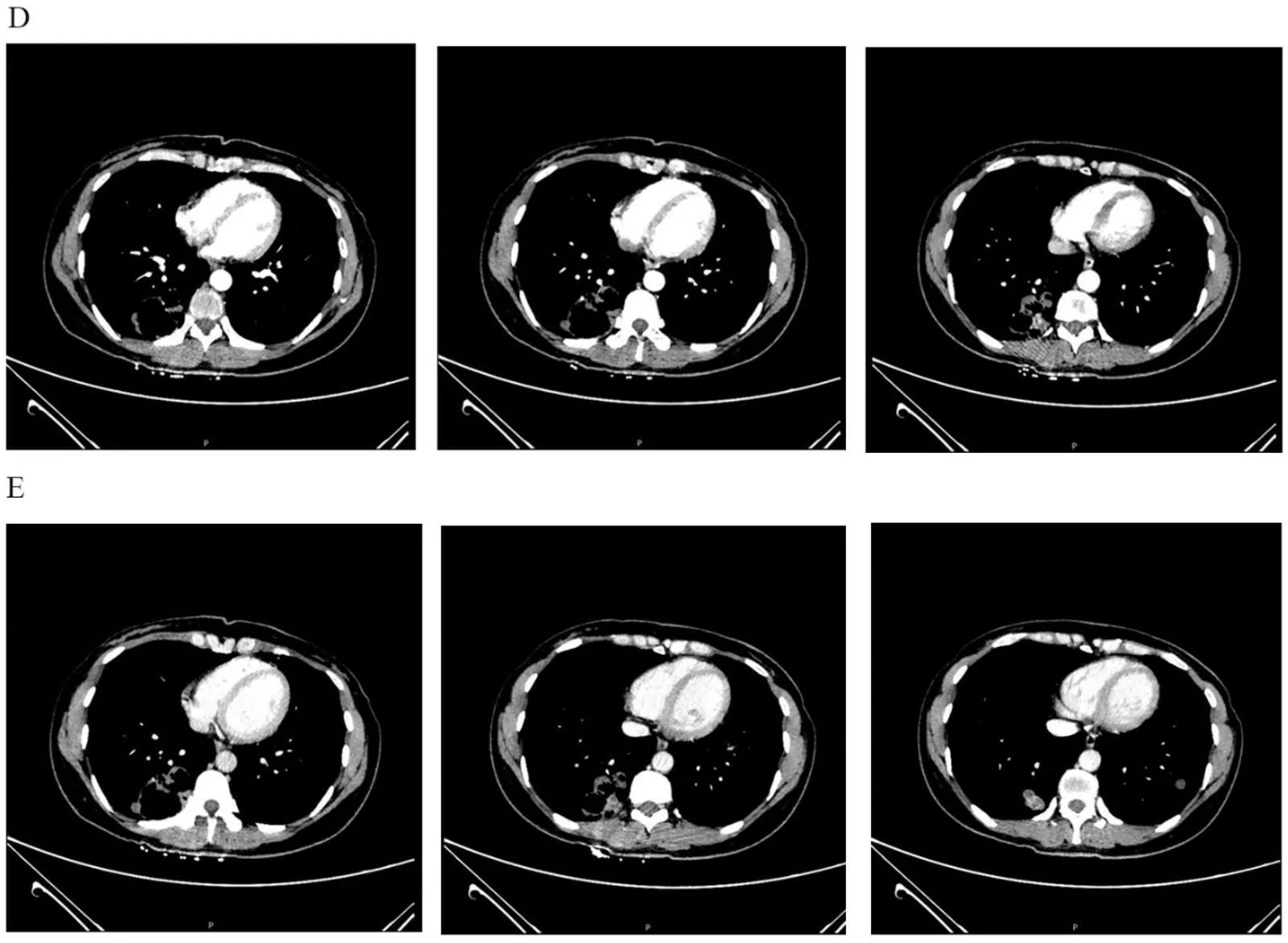
Lung CT scanning revealed a lesion in the right lower lobe. A thick-walled cavity is observed in the posterior basal segment of the right lower lobe. The wall is uneven in texture and measures approximately 57 × 40 mm (the enhanced wall shows mild enhancement). Multiple nodules are present around it, with the largest one measuring about 23 × 15 mm: (**A**) the CT plain scan image—mediastinum window image; (**B**) CT plain scan—lung window; (**C**) the arterial phase of the CT enhanced scan; (**D**) the enhanced CT scan in the venous phase; (**E**) CT reconstruction.

**Figure 2:**
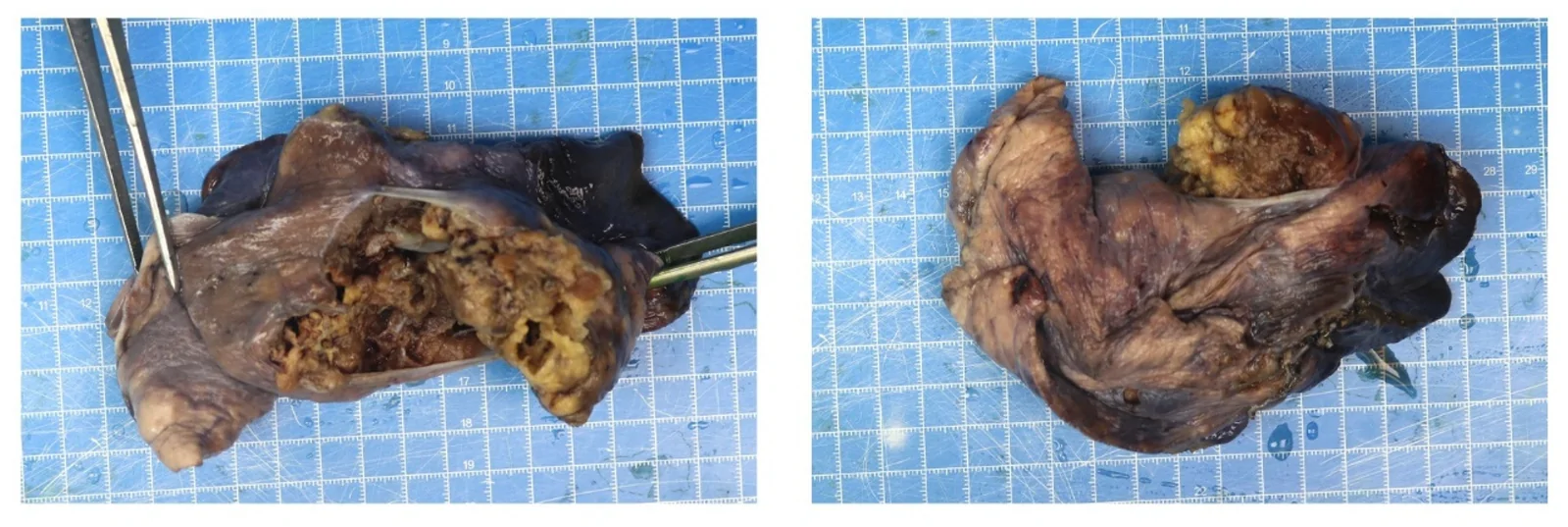
Surgical specimen of the right lower lobe.

**Figure 3:**
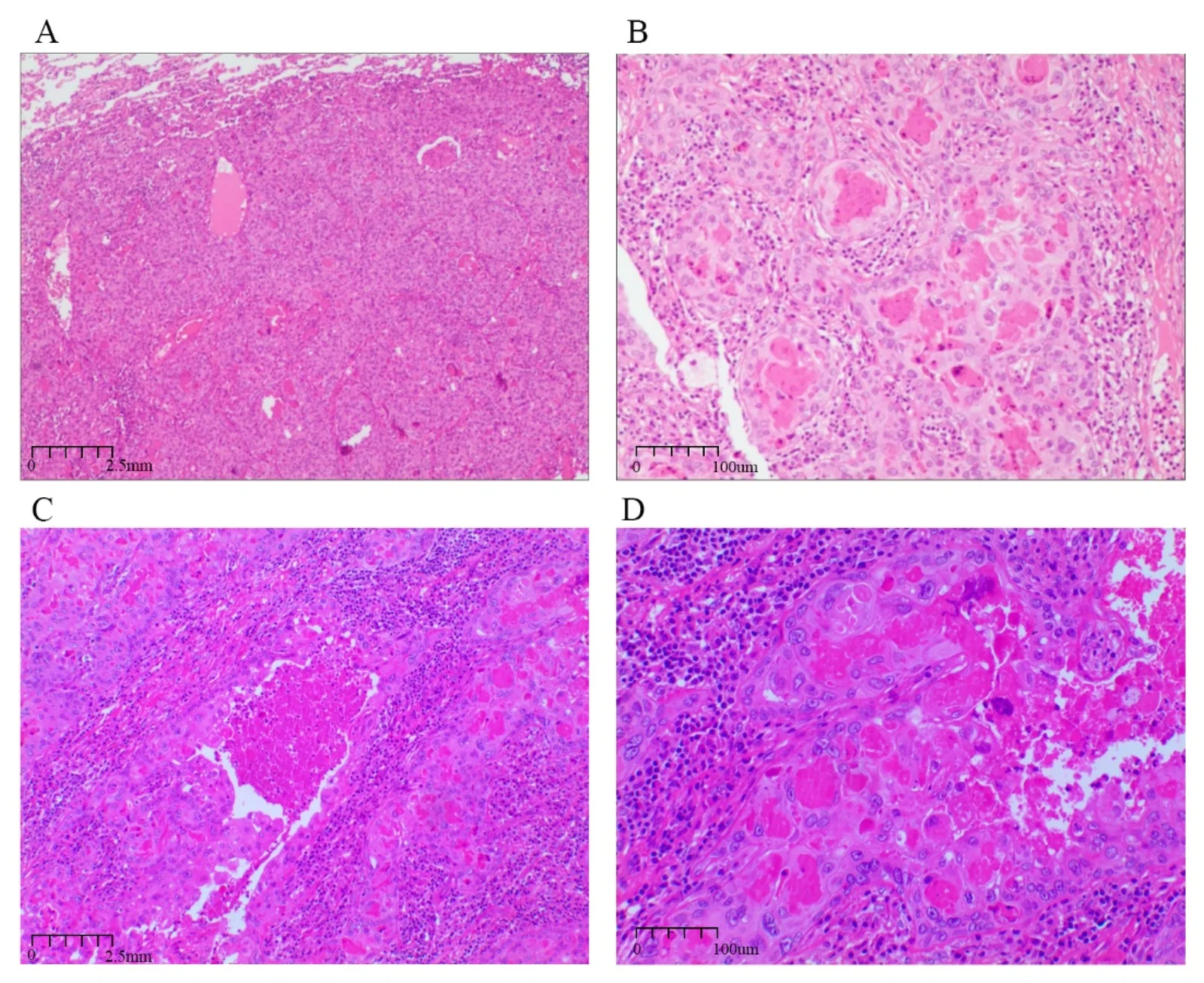
Epithelioid trophoblastic tumor lung lesion. Hematoxylin and eosin staining of the lung lesion at low (**A**,**C**) and high (**B**,**D**) magnification. Scale bars: 2.5 mm (**A**,**C**) and 100 µm (**B**,**D**). Hematoxylin and eosin (H&E) staining was performed using a Harris hematoxylin solution (Solarbio, Beijing, China) and eosin Y solution (Solarbio, Beijing, China). Immunohistochemical staining was performed using the EnVision FLEX detection system (Dako, Glostrup, Denmark) according to the manufacturer’s instructions.

**Figure 4:**
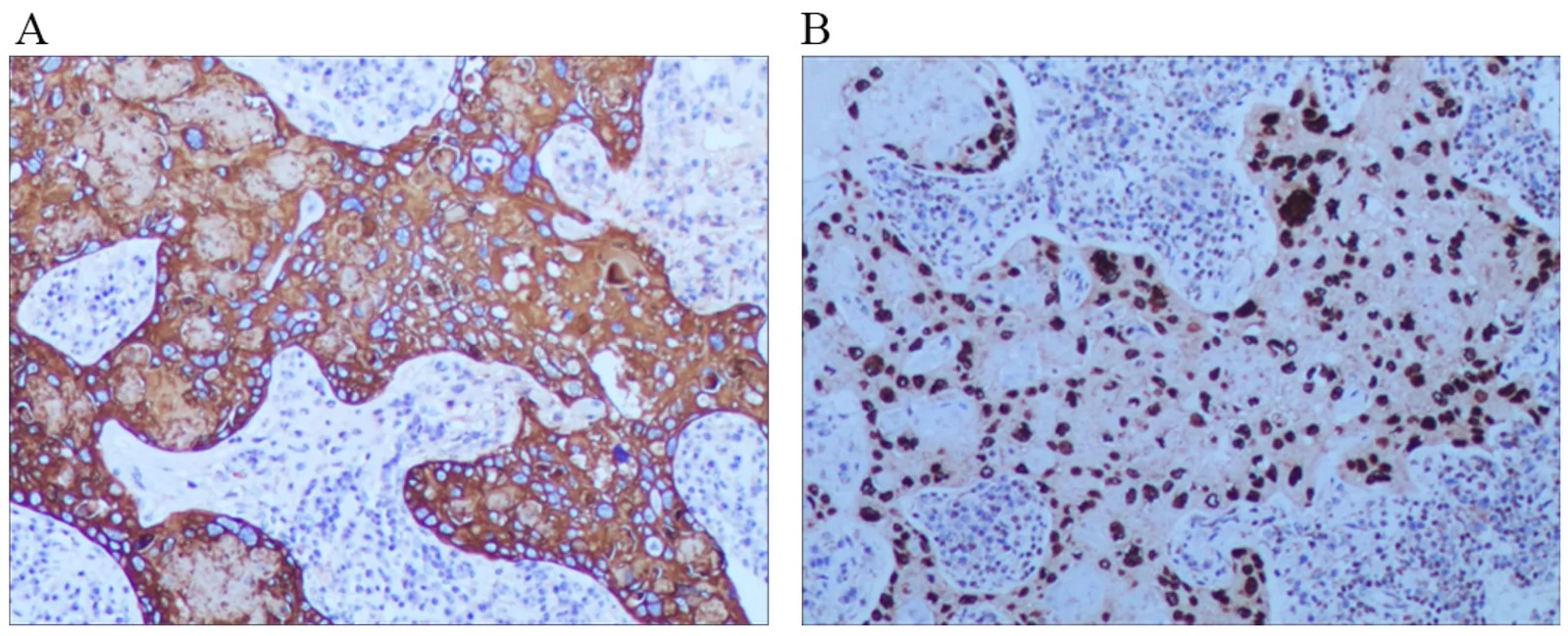

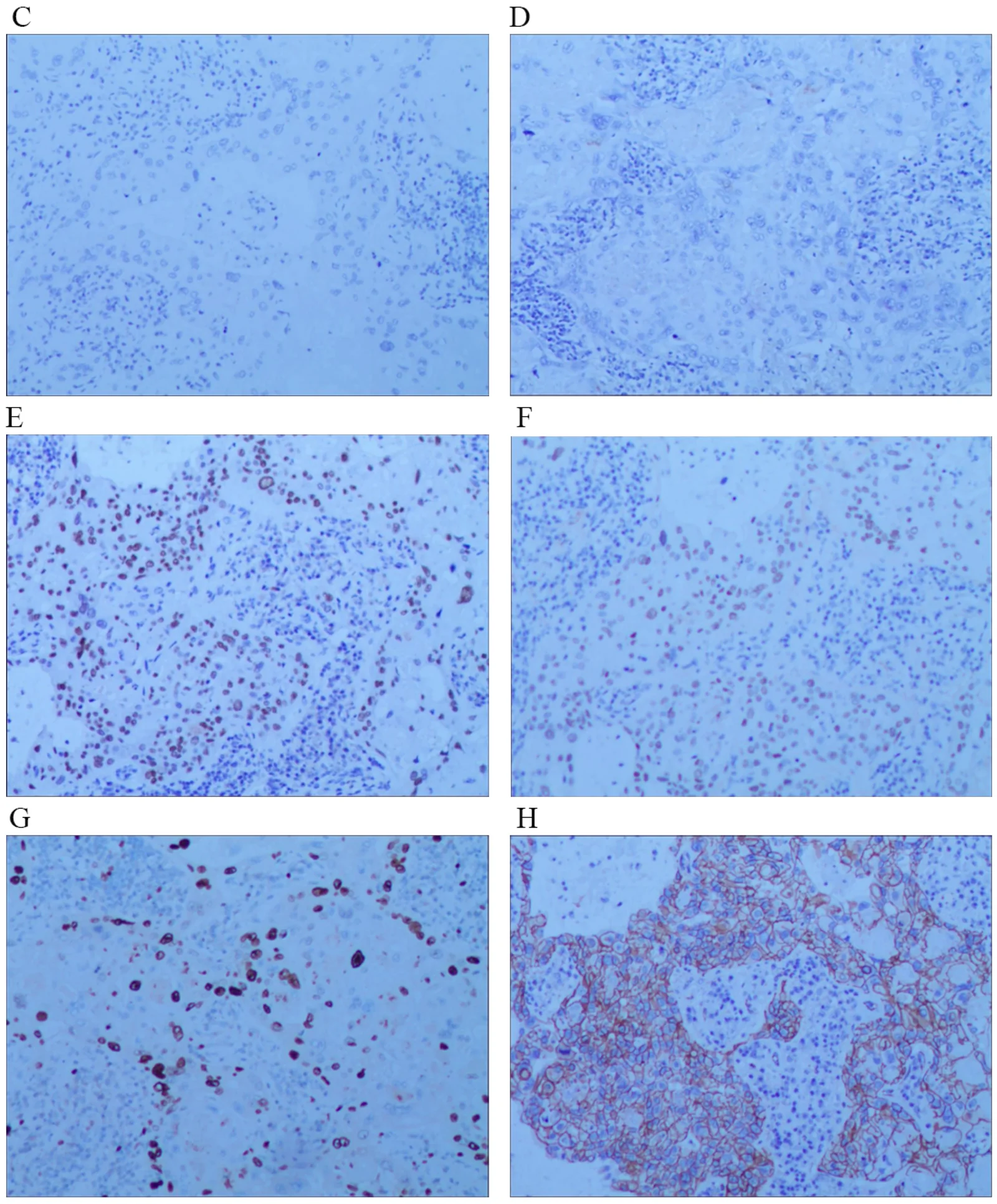
Immunohistochemical staining demonstrated positive expression of the following markers. Immunohistochemical staining (×20): (**A**) CK7 (+); (**B**) GATA-3 (+); (**C**) hPL (+, focal positivity in multinucleated cells); (**D**) β-hCG (+, focal positivity in multinucleated cells); (**E**) P63 (+); (**F**) P40 (+); (**G**) Ki-67 (+) 30%; (**H**) β-catenin (+). Immunohistochemical staining was performed on formalin-fixed, paraffin-embedded tissue sections using the EnVision two-step method. The primary antibodies used in this study were as follows: CK7 (clone OV-TL 12/30, Dako, Glostrup, Denmark), GATA-3 (clone L50-823, Cell Marque, Rocklin, CA, USA), human placental lactogen (hPL, clone PL-1, Dako, Glostrup, Denmark), β-human chorionic gonadotropin (β-hCG, clone Polyclonal, Dako, Glostrup, Denmark), p63 (clone 4A4, Dako, Glostrup, Denmark), P40 (clone BC28, Biocare Medical, Pacheco, CA, USA), β-catenin (clone β-catenin-1, Dako, Glostrup, Denmark), and Ki-67 (clone MIB-1, Dako, Glostrup, Denmark).

A month later, the patient sought care at our hospital, where a computed tomography (CT) scan revealed a 57 × 40 mm lesion in the right lower lobe (Figure 1). Pelvic contrast-enhanced magnetic resonance imaging (MRI) revealed no evidence of an intrauterine, cervical, or adnexal mass, nor any abnormal myometrial enhancement or invasion. Gynecological examination, including speculum examination and bimanual palpation, showed a normal-appearing cervix with no visible or palpable lesions. These findings, combined with the previous negative transvaginal ultrasonography and endometrial curettage, provided no evidence of an identifiable uterine or cervical primary. At admission, her serum β-hCG concentration was 993 mIU/mL. Transvaginal ultrasonography revealed an endometrial thickness of 12 mm. The patient denied experiencing respiratory symptoms, including cough, chest pain, and hemoptysis. Lung cancer biomarkers were within normal reference ranges, and the tuberculin skin test yielded a negative result. Differential diagnoses included persistent ectopic pregnancy and quiescent gestational trophoblastic disease. Staging and risk assessment: According to the FIGO 2000 staging and modified WHO prognostic scoring system for gestational trophoblastic neoplasia, the patient was staged as follows: age 39 years (score 0); antecedent term pregnancy (score 2); interval from index pregnancy 36 months (score 4); pretreatment serum β-hCG 978–1100 mIU/mL (score 1); largest tumor size 57 mm (score 2); site of metastases: lung (score 0); number of metastases: 1 (score 1); and previous failed single-agent chemotherapy (methotrexate, score 2). The total WHO prognostic score was 12, classifying this as ultra-high-risk GTN. The FIGO anatomical stage was III. The final stage and score designation was Stage III:12. The multidisciplinary team, involving experts from gynecology and thoracic surgery, deliberated on treatment options. Ultimately, the patient underwent a successful right lower lobe resection and lymph node dissection by thoracoscopic surgical method (Figure 2), resulting in a rapid normalization of the β-hCG level. Final histopathological analysis confirmed the diagnosis of pulmonary epithelioid trophoblastic tumor (ETT). Histopathological and immunohistochemical analyses of the lung tissue confirmed ETT, showing positivity for Ki-67 (30%, assessed in hotspot areas), P63 (diffuse nuclear), CK7, β-catenin, and GATA-3, with focal hPL and HCG expression confined to scattered multinucleated syncytiotrophoblast-like giant cells. The tumor was negative for CK5/6, TTF-1, P16, and Muc4 (Figure 3 and Figure 4). Three regional lymph node groups showed no metastatic involvement. PETT is a very rare gestational trophoblastic disease originating from intermediate trophoblastic cells of the chorion laeve. In light of absent uterine involvement and the patient’s desire for fertility preservation, hysterectomy was not performed. She received 3 cycles of combination EMA/Co chemotherapy (etoposide, methotrexate, and actinomycin D, followed by cyclophosphamide and vincristine), and the serum β-hCG level dropped to normal (<5 mIU/mL). At the 3-year posttreatment follow-up, the patient reported regular menstrual cycles, with serum β-hCG levels consistently within normal limits. Serum β-hCG was measured weekly during chemotherapy and for the first 3 months after completion of treatment, then every 2 weeks for the subsequent 3 months, monthly during the second year, and quarterly thereafter. Chest CT and pelvic ultrasound were performed every 3 months in the first year, every 6 months in the second year, and annually from the third year onward. Regular imaging examinations, including chest CT and pelvic ultrasound, did not reveal any signs of recurrence. During the 3-year follow-up period, the patient only experienced some digestive tract discomfort symptoms during the postoperative chemotherapy process, such as nausea, vomiting, loss of appetite, etc. These adverse toxic effects were treated actively, allowing the patient to recover and stabilize. A timeline of key clinical events and β-hCG values is provided in Supplementary Table S2, which lists all major clinical events (surgeries, chemotherapy cycles, and imaging studies) with corresponding dates and β-hCG levels.

## 3. Literature Search Strategy

To place our case in context and to pool previously reported experience, we systematically searched PubMed, Web of Science, and CNKI from inception through March 2026, using the terms (“epithelioid trophoblastic tumor” OR “ETT”) AND (“lung” OR “pulmonary”) AND (“case report” OR “case series”). The reference lists of retrieved articles and relevant reviews were hand-searched for additional studies. Eligible studies (i) reported histopathologically confirmed ETT; (ii) documented pulmonary involvement as the presenting site or as isolated metastasis without a detectable uterine primary; (iii) provided extractable case-level data, including at minimum the serum β-hCG concentration and treatment modality; and (iv) were published in English or Chinese. Studies were excluded if they were (i) duplicate publications; (ii) reviews, editorials, or conference abstracts without extractable individual case data; or (iii) reports based on overlapping patient cohorts.

## 4. Discussion

Pulmonary trophoblastic neoplasms constitute a rare group of gestational and non-gestational malignancies, including choriocarcinoma, ETT, and placental-site trophoblastic tumor. Among these, primary pulmonary choriocarcinoma and primary pulmonary ETT are the most commonly encountered subtypes, exhibiting a marked predilection for women of reproductive age; however, isolated cases have also been reported in male patients. In this report, we describe a diagnostically challenging case of pulmonary ETT that exemplifies the clinical complexity and diagnostic pitfalls associated with this entity. The patient was initially misdiagnosed with ectopic pregnancy and experienced a prolonged diagnostic delay before the correct diagnosis was ultimately established. This case highlights several critical clinical lessons: the importance of considering atypical trophoblastic lesions in the differential diagnosis; the indispensable role of comprehensive histopathological and immunohistochemical evaluation; and the necessity of maintaining a high index of suspicion for extrauterine trophoblastic tumors in patients presenting with pulmonary lesions and elevated serum β-hCG levels—even in the absence of a documented gestational history.

The preoperative diagnosis of pulmonary ETT remains challenging, owing to its nonspecific clinical presentation, variable serum β-hCG levels, and histopathological resemblance to more common pulmonary neoplasms. To address this diagnostic dilemma, we summarized the immunophenotypic and clinical characteristics of PETT in comparison with its principal mimics and with other trophoblastic entities (Table 1).

### 4.1. Quantitative Synthesis of Reported Cases

Thirty cases of pulmonary ETT without demonstrable uterine involvement were identified, including the present case (Supplemental Table S1). The median age at diagnosis was 33 years (range, 23–49). Fourteen patients (46.7%) presented with abnormal vaginal bleeding and eight (26.7%) with an incidental lesion on imaging. A term pregnancy was the antecedent gestation in 15 cases (50.0%), at a median interval of 36 months (range, 3–96). Pretreatment serum β-hCG ranged from 0.7 to 2204 mIU/mL (median, 304), and 22 patients (73.3%) had levels below 1000 mIU/mL. All 30 patients underwent surgical resection—VATS in 22 (73.3%)—and 18 (60.0%) received adjuvant chemotherapy, predominantly EMA/CO (*n* = 8) or EMA/EP (*n* = 7). After a median follow-up of 24 months (range, 3–90), 28 patients (93.3%) were alive without evidence of disease; both adverse outcomes followed incomplete treatment or suboptimal resection, indicating that complete surgical resection is the mainstay of cure.

### 4.2. The Limitations and Value of Serum β-hCG Diagnosis

The patient’s initial β-hCG level of 978 mIU/mL, rising to 1100 mIU/mL after failed methotrexate therapy, falls within an intermediate range that is characteristic of ETT and distinct from choriocarcinoma, where β-hCG typically exceeds 10,000 mIU/mL. This relatively modest elevation creates a diagnostic trap: it is high enough to suggest pregnancy-related pathology, yet low enough that the possibility of ectopic pregnancy dominates the differential diagnosis, as occurred in this case. Three recent cases followed nearly identical trajectories. Ma et al. [4] reported a 29-year-old woman with isolated pulmonary ETT presenting with β-hCG of 659 mIU/mL, initially managed as suspected pregnancy before VATS lobectomy confirmed the diagnosis. Slattery et al. [13] described a 34-year-old woman whose β-hCG of 706 mIU/mL persisted for over three years through empirical methotrexate therapy, ultimately found to have a left lower lobe ETT resected with sustained 18-month remission. Zhang et al. [11] reported another case of primary pulmonary ETT in a patient with β-hCG of approximately 800 mIU/mL, similarly misdiagnosed initially. In contrast, Li et al. documented uterine ETT with synchronous lung metastases where β-hCG surged from 1031 to 2034 mIU/mL, requiring hysterectomy and multi-agent chemotherapy, yet still recurred within 2.5 months [6]. Collectively, these cases demonstrate that the absolute β-hCG value alone cannot reliably distinguish extrauterine ETT from ectopic pregnancy. Rather, the critical red flag that should prompt extra-pelvic investigation lies in the atypical dynamics of β-hCG, namely its failure to return to normal levels following methotrexate treatment and after adnexal pathology has been ruled out. To enhance diagnostic specificity, it is recommended to conduct continuous β-hCG testing to monitor the dynamic changes over time rather than relying on a single point value for assessment. Given the expression of supplementary biomarkers such as human placental lactogen and inhibin A in trophoblastic tumors, it has been proposed that this could improve diagnostic accuracy, but its clinical utility still awaits pathological confirmation [14].

### 4.3. Histogenesis and Molecular Characterization of Pulmonary ETT

In the present case, no evidence of a primary genital tract malignancy was identified on multimodality workup: transvaginal ultrasonography, contrast-enhanced pelvic MRI, and gynecological examination disclosed no uterine, cervical, or adnexal lesions. Endometrial thickness measured 12 mm, marginally greater than the 10 mm recorded initially; in the absence of any corresponding MRI abnormality, this was attributed to the estrogen- and progesterone-driven hormonal milieu secondary to persistent trophoblastic β-hCG production, rather than to intrauterine disease. Although microscopic uterine disease cannot be entirely excluded, these concordant negative findings strongly support the lung as the only clinically detectable site of involvement.

The occurrence of primary ETT in the lung without a detectable uterine lesion raises fundamental questions about its histogenesis. Several hypotheses merit consideration. First, the tumor may originate from ectopic chorionic-type intermediate trophoblasts deposited in the pulmonary microvasculature during a prior pregnancy through trophoblastic deportation—a phenomenon well documented in normal gestation where trophoblastic fragments embolize to the lungs and are usually cleared [15]. Under permissive conditions (immunological, genetic, or hormonal), these dormant cells may undergo malignant transformation after a latency period extending years. ETT is known to follow a term pregnancy in 60–70% of cases, with a latency that can exceed a decade [3]. Second, short tandem repeat (STR) genotyping has emerged as an indispensable molecular tool to resolve the gestational origin of these tumors: by identifying unique paternal allelic patterns in tumor tissue, it can definitively differentiate ETT from somatic carcinoma mimics such as squamous cell carcinoma or primary lung adenocarcinoma [16]. Although STR analysis was not performed in this case, we recommend its incorporation into the standard diagnostic workup for all suspected extrauterine ETT presentations. Third, transcriptomic profiling has revealed that ETT frequently exhibits PI3K-Akt-mTOR pathway activation and PTEN loss, which may contribute to its distinctive biology—slower growth than choriocarcinoma yet notable chemoresistance [7]. Additionally, strong PD-L1 expression has been documented in ETT, laying a molecular foundation for exploring immune checkpoint inhibitors in chemorefractory cases [17,18].

### 4.4. Proposed Diagnostic Algorithm for Extrauterine ETT

**Figure 5:**
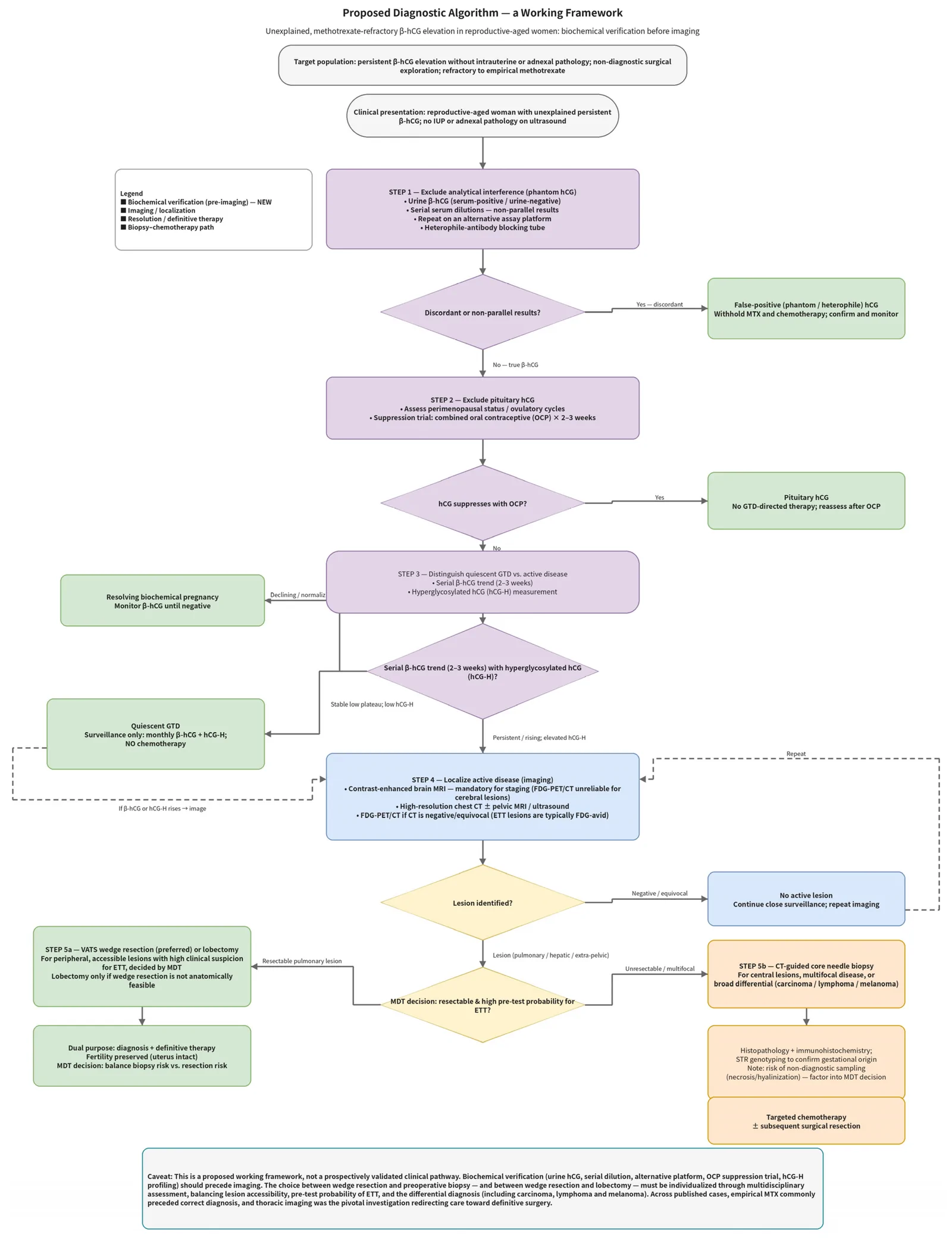
Proposed Diagnostic Algorithm—A Working Framework. This flowchart illustrates a structured approach to the reproductive-aged woman with persistent β-hCG elevation, no demonstrable intrauterine or adnexal pathology, and failure of empirical methotrexate therapy. Biochemical verification (β-hCG, serial dilution, alternative-platform assay, OCP suppression trial, and hyperglycosylated-β-hCG profiling) should be completed before imaging to exclude analytical interference, pituitary β-hCG, and quiescent gestational trophoblastic disease. When a solitary pulmonary lesion is identified, the choice between diagnostic wedge resection and preoperative CT-guided biopsy should be made through multidisciplinary assessment, weighing lesion accessibility, the pre-test probability of ETT, and the differential diagnosis (which includes primary lung carcinoma, lymphoma, melanoma, and metastatic carcinoma). This algorithm is intended as a descriptive proposal based on the authors’ experience and the available literature; it has not been prospectively validated and should not replace individualized, multidisciplinary clinical decision-making. ETT, epithelioid trophoblastic tumor; OCP, combined oral contraceptive; β-hCG-H, hyperglycosylated human chorionic gonadotropin; VATS, video-assisted thoracoscopic surgery; STR, short tandem repeat genotyping.

The clinical impact of this case extends beyond its rarity to inform a reproducible diagnostic approach. We propose a structured algorithm for reproductive-aged women with unexplained, methotrexate-refractory β-hCG elevation in the absence of intrauterine or adnexal pathology (Figure 5). The guiding principle is that biochemical verification must precede imaging, thereby avoiding unnecessary radiation and surgical intervention for conditions that do not require GTN-directed therapy. The algorithm proceeds in five phases: (i) Step 1: Analytical interference must be ruled out—often referred to as phantom or heterophile antibody-mediated β-hCG. This is accomplished through a panel of inexpensive tests: urine β-hCG (a serum-positive/urine-negative pattern is highly suggestive of interference), serial serum dilutions demonstrating non-parallel results, repeat measurement on an alternative assay platform, and, where available, heterophile-antibody blocking tubes. (ii) Step 2: Pituitary-derived β-hCG should be excluded, particularly in perimenopausal women or those with irregular ovulatory cycles. A 2–3-week trial of combined oral contraceptive suppression typically reduces pituitary β-hCG to undetectable levels. (iii) Step 3: Serial β-hCG monitoring over 2–3 weeks, ideally paired with hyperglycosylated β-hCG measurement, helps distinguish resolving biochemical pregnancy, quiescent GTD, and truly active disease requiring further investigation. (iv) Step 4: Once active disease is confirmed biochemically, localization imaging is obtained. Contrast-enhanced brain MRI is included at this stage for formal staging, because FDG-PET/CT has poor sensitivity for cerebral lesions and because the detection of brain involvement upstages the disease to FIGO Stage IV and alters management [19,20]. Perform high-resolution chest CT ± pelvic MRI to identify occult pulmonary, hepatic, or extra-pelvic lesions. When no active lesion is identified, close surveillance with repeat imaging is appropriate. (v) Step 5: When a solitary pulmonary lesion is identified, decide on the initial tissue-acquisition strategy through multidisciplinary assessment by gynecologic oncologists, thoracic surgeons, interventional radiologists, and pathologists. The choice between diagnostic wedge resection and preoperative CT-guided core-needle biopsy depends on lesion accessibility, the clinical pre-test probability of ETT, and the breadth of the differential diagnosis. Wedge resection (VATS) is preferred for peripheral, anatomically favorable lesions in patients with a high clinical suspicion for ETT (e.g., persistent or rising β-hCG with no uterine/adnexal source, typical FDG-avidity), because it provides both a definitive diagnosis and complete resection in a single procedure [21]. Lobectomy is reserved for lesions not amenable to wedge resection. CT-guided biopsy is appropriate for centrally located lesions, multifocal disease, or cases in which the differential diagnosis prominently includes non-gestational entities such as primary lung carcinoma, lymphoma, melanoma, or metastatic carcinoma—because a tissue diagnosis prior to major surgery may substantially alter management. The known risk of non-diagnostic sampling on needle biopsy due to ETT’s heterogeneous morphology, hyalinized stroma, and necrotic components should be factored into the multidisciplinary discussion but should not be considered a contraindication to biopsy when clinically indicated. For unresectable or multifocal disease, obtain tissue via CT-guided biopsy for histopathological and immunohistochemical analysis, with STR genotyping to confirm gestational origin [16]. It should be emphasized that this algorithm represents a proposed working framework, derived from the present case and from the limited published experience, and is not a prospectively validated clinical pathway. Management decisions must always be individualized and made jointly by a multidisciplinary team and the patient, with full informed consent regarding diagnostic uncertainty, procedural risks, and fertility implications. Fertility preservation is also a critical consideration, as these patients are predominantly of reproductive age. In the absence of demonstrable uterine involvement, lobectomy preserves the uterus and ovarian function, enabling the possibility of future pregnancy through assisted reproductive technology (IVF), which is a viable option given that the patient’s ovaries were not affected. This highlights the unique advantage of prompt diagnosis in facilitating fertility-sparing management, although patients must be counseled that natural conception is no longer possible following bilateral salpingectomy.

### 4.5. Microscopic Morphology and Immunohistochemical Characteristics

The histological features of ETT are characterized by relatively uniform populations of polygonal epithelioid trophoblastic cells arranged in nests or cords. These cells exhibit abundant eosinophilic cytoplasm and distinct cell borders, typically accompanied by geographic or map-like necrosis and hyaline or glassy degeneration. Unlike choriocarcinoma, ETT lacks the extensive hemorrhage and necrosis characteristic of that tumor type, which aids in its histopathological differentiation. The tumor cells generally demonstrate a monomorphic appearance with moderate nuclear atypia and mitotic activity but lack the marked pleomorphism observed in other trophoblastic neoplasms. In 2021, Oliver and colleagues first identified and reported the LPCAT1-TERT fusion gene as a recurrent and specific molecular marker in ETT, driving TERT overexpression through genomic deletion and promoting cellular proliferation, thereby providing new directions for precision diagnosis and targeted therapy in this rare chemotherapy-resistant tumor [22]. Immunohistochemically, ETT tumor cells consistently express epithelial markers such as cytokeratin 7 (CK7) and epithelial membrane antigen (EMA), as well as inhibin-α, reflecting their trophoblastic origin. The expression of β-hCG is typically weak or focal, which distinguishes ETT from choriocarcinoma, where β-hCG expression is diffuse and strong [23]. Notably, a study analyzing the morphology, immunohistochemistry, and next-generation sequencing of 9 cases of PSTT and 4 cases of ETT demonstrated that combined immunohistochemical detection of p63, hPL, and CD146 can effectively differentiate ETT from PSTT. Specifically, ETT exhibits strong nuclear positivity for p63, a marker valuable for distinguishing it from other trophoblastic tumors and carcinomas. Compared to PSTT and other placental site trophoblastic lesions, ETT demonstrates enhanced expression of human placental lactogen and Mel-CAM (CD146). The study also identified that the interval from the preceding pregnancy to diagnosis (>48 months), deep myometrial invasion, mitotic count, and Ki-67 proliferation index are important parameters for predicting metastasis, although no single indicator serves as an absolute predictor [24]. This immunohistochemical profile is critical for accurate diagnosis, particularly given the morphological overlap with squamous cell carcinoma and other epithelial malignancies. The combination of these histological and immunohistochemical features provides a robust framework for identifying ETT and distinguishing it from other gestational trophoblastic neoplasms and morphologically similar tumors [25,26]. The diagnosis of primary pulmonary ETT requires the exclusion of various tumors with similar histological and immunophenotypic features, including non-small cell lung cancer (NSCLC), squamous cell carcinoma, large cell carcinoma, lymphoma, malignant melanoma, and pulmonary metastasis of uterine primary ETT. Therefore, we summarized the main immunohistochemical markers that aid in the differential diagnosis, as shown in Table 2. 

Of note, the Ki-67 index of 30% observed in this case should not be interpreted as evidence of a choriocarcinomatous component. Choriocarcinoma was excluded on several grounds. Morphologically, the tumor lacked the characteristic biphasic arrangement of cytotrophoblast and syncytiotrophoblast, as well as the extensive hemorrhage and necrosis typical of choriocarcinoma, and instead consisted of a monophasic population of intermediate trophoblasts with epithelioid morphology. Immunophenotypically, the tumor cells showed diffuse nuclear p63 positivity—p63 being characteristically negative in choriocarcinoma—while the Ki-67 index of 30% remained well below the >50–70% range generally observed in choriocarcinoma. Finally, the scattered multinucleated cells expressing focal β-hCG and hPL represent a well-documented finding in a subset of ETTs and do not indicate a mixed choriocarcinomatous component.

### 4.6. Fertility Preservation and Multidisciplinary Management

ETT predominantly affects women of reproductive age, and the majority of patients have not yet completed their family planning at the time of diagnosis. Achieving effective tumor control while preserving fertility constitutes a core challenge in clinical decision-making. Unlike choriocarcinoma, a GTN subtype that is highly sensitive to chemotherapy, ETT is relatively chemo-resistant, and total hysterectomy is widely regarded as the definitive treatment for localized disease. However, for early-stage patients with a strong desire for fertility preservation, fertility-sparing treatment strategies are gradually gaining attention. Currently, the evidence supporting fertility-sparing treatment in ETT is derived primarily from case reports and small case series. A multicenter retrospective study involving 31 ETT patients reported that 5 Stage I patients underwent fertility-sparing treatment, among whom 1 achieved a full-term delivery; however, another 23-year-old patient who refused hysterectomy died of disease recurrence. The same study observed that patients with isolated pulmonary ETT tended to be younger (median 30.5 vs. 41 years) and to have smaller lesions (median 2.4 vs. 4.8 cm) compared with those with other metastatic disease, and none of the eight patients in this subgroup died of disease [27]. These descriptive findings are consistent with the hypothesis that isolated pulmonary ETT may represent a clinically favorable subset; however, they derive from a small, retrospectively defined subgroup and should be interpreted cautiously. Huang et al. reported a 32-year-old patient with bilateral pulmonary ETT lesions who underwent two wedge resections and segmentectomy without hysterectomy; her menstrual cycles resumed postoperatively, and no recurrence was observed during 12 months of follow-up [28]. These cases suggest that, for patients with completely resectable localized ETT, fertility-sparing surgery combined with adjuvant chemotherapy may represent a viable treatment strategy.

Nevertheless, the decision to pursue fertility-sparing treatment must be made with exceptional caution. The literature emphasizes that data on fertility-sparing treatment in ETT remain exceedingly limited, and this option should be offered judiciously. Key decision-making factors include tumor stage, number of lesions, resectability, and patient age. A multicenter retrospective analysis of 31 ETT patients identified a high metastatic burden as a strong adverse prognostic indicator: on multivariate analysis, ≥3 metastases was associated with poorer overall and recurrence-free survival, and ROC analysis identified a cut-off of ≥3 metastases as the optimal discriminator (AUC 0.86) [27]. Importantly, these estimates derive from a small single-institution (three-center) series and are associated with wide confidence intervals—illustrated by the only reported interval, for the unadjusted association with OS, of HR 26.26 (95% CI 2.96–233.0)—and should therefore be viewed as hypothesis-generating rather than as a validated threshold for clinical decision-making. Accordingly, fertility-preserving approaches, when considered, are best reserved for patients with localized disease and a low metastatic burden—for example, Stage I disease or isolated, fully resectable pulmonary lesions—and must always be balanced against the risk of recurrence, which is not negligible. The available evidence is insufficient to define a safe cut-off on the basis of the number of metastases alone. The impact of chemotherapeutic agents on ovarian function must also be factored into treatment planning. Alkylating agents (such as cyclophosphamide) and etoposide-containing regimens can impair ovarian function in a dose-dependent manner, whereas methotrexate and dactinomycin exert comparatively less gonadotoxic effects [29]. In the present case, the patient was counseled to use reliable barrier contraception for at least 12 months after completion of EMA/CO chemotherapy and to defer pregnancy planning until her β-hCG had remained undetectable for a full year and she had completed the intensive phase of surveillance. At last follow-up, the patient reported regular menstrual cycles and had used contraception consistently as advised. She had not attempted conception during the 3-year follow-up period.

The critical role of a multidisciplinary team (MDT) in managing such complex cases cannot be overstated. In our patient’s case, collaboration between gynecologists and thoracic surgeons was essential in re-evaluating clinical data, identifying the pulmonary lesion, and developing an effective treatment plan. The MDT approach ensures that all diagnostic possibilities are thoroughly explored and that the most appropriate patient-specific treatment strategy is selected. Ultimately, surgical resection has proven to be the cornerstone of both diagnosis and treatment. The decision to perform a right lower lobectomy was transformative. This procedure achieved a complete gross resection of the tumor, which was corroborated by the subsequent rapid normalization of serum β-hCG levels, a key biomarker for disease burden. Histopathological analysis of the resected specimen provided a definitive diagnosis, revealing characteristic features of ETT: nests of epithelioid trophoblastic cells accompanied by eosinophilic debris and geographic necrosis, supported by an immunohistochemical profile typically positive for p63, cytokeratin, and focal β-hCG. For localized chemoresistant lesions such as this one, surgical resection is not only the preferred diagnostic modality but also serves as the primary curative treatment.

### 4.7. Limitations of This Study

This study is subject to several limitations. The absence of STR genotyping in this case represents a missed opportunity to definitively exclude the possibility of metastatic ETT from an occult uterine primary below imaging detection thresholds. Furthermore, ETT is known for its indolent growth and capacity for delayed relapse, with recurrences reported up to several years after initial treatment. Therefore, extended surveillance beyond 5–10 years is warranted [30,31]. An unresolved clinical question is the role of adjuvant chemotherapy after R0 resection of isolated pulmonary ETT. Slattery et al. reported a case of isolated pulmonary Stage III ETT that achieved sustained remission with surgery alone, whereas Li et al. observed recurrence when systemic treatment was suboptimal. In the present case, remission was achieved following complete surgical resection combined with adjuvant EMA/CO chemotherapy, making it difficult to disentangle the relative contributions of surgery and systemic therapy. Multicenter registry-based studies that prospectively collect standardized data on treatment regimens, β-hCG kinetics, and long-term outcomes are urgently needed to establish evidence-based guidelines for this rare entity. We acknowledge that brain imaging was not performed as part of the initial staging workup. This represents a significant limitation, as the presence of brain metastases would have upstaged the disease to FIGO Stage IV and altered the therapeutic approach. In addition to this, CD146 (Mel-CAM) immunostaining was not performed. While a combined panel of p63, hPL, and CD146 is useful for distinguishing ETT from PSTT, the diagnosis of ETT in this case was based on characteristic morphological features (nested growth with geographic necrosis and hyaline material deposition) and a supportive immunohistochemical profile (diffuse p63 positivity, CK7, GATA-3, and β-catenin positivity, with hPL and HCG expression confined to scattered multinucleated cells). The absence of CD146 is acknowledged as a limitation; however, the morphological and immunohistochemical features, taken together, were considered diagnostic of ETT.

A 39-year-old woman with pulmonary ETT and no demonstrable uterine disease achieved sustained three-year remission after right lower lobectomy and three cycles of adjuvant EMA/CO, with uterine and ovarian function preserved. Her diagnostic odyssey—misdiagnosis as ectopic pregnancy, failed methotrexate, non-diagnostic salpingectomy—epitomizes a pattern repeatedly reported for this rare entity. Three observations bear emphasis. First, a pulmonary lesion with elevated β-hCG and negative pelvic findings should trigger consideration of extrauterine trophoblastic disease before additional empirical therapy is given. Second, complete surgical resection of a resectable lesion accomplishes both tissue diagnosis and local control and, in this case, was followed by prompt β-hCG normalization. Third, fertility-sparing management can succeed when no uterine primary is found, though patient selection remains critical.

Beyond this single case, two points merit comment. The 93% disease-free rate seen across 30 published resected pulmonary ETT cases aligns with the view that surgery is central to curative management and supports the inclusion of STR genotyping in the diagnostic workup to confirm gestational origin against somatic mimics. Extended surveillance beyond standard GTN follow-up is reasonable given the indolent course and documented late relapses. In conclusion, the stepwise pre-imaging biochemical algorithm we outline (Figure 5) is offered as a practical framework to reduce unnecessary intervention, not as a validated pathway. Prospective registry data will be needed to move beyond hypothesis-generating case-level evidence.

## Figures and Tables

**Table 1:** Key clinical and pathological features distinguishing pulmonary epithelioid trophoblastic tumor (ETT) from primary pulmonary choriocarcinoma (PPC) and gestational choriocarcinoma (GC). The deportation hypothesis we invoke for ETT is conceptually analogous to the gestational subtype of primary pulmonary choriocarcinoma, not the non-gestational form.

Feature	Gestational Choriocarcinoma (GC)	Primary Pulmonary Choriocarcinoma (PPC)	Pulmonary Epithelioid Trophoblastic Tumor (ETT)
Origin	Malignant trophoblastic neoplasm arising from uterine or ectopic pregnancy	Ectopic germ cell/non-gestational origin within the lung	Epithelioid trophoblastic tumor arising from intermediate trophoblastic cells of the chorion laeve in the lung
Associated Pregnancy Event	Yes (hydatidiform mole, abortion, term delivery, etc.)	Gestational and non-gestational (males/postmenopausal females)	Gestational (women of reproductive age with pregnancy history) or non-gestational
Affected Population	Women of reproductive age	Both males and females	Women of reproductive age
Serum β-hCG Level	Markedly elevated (often > 10,000 mIU/mL)	Markedly elevated (similar to GC)	Mild to moderately elevated (or within normal range)
Immunohistochemistry	- β-hCG: Strongly positive (syncytiotrophoblasts) - p63: Negative or focal weak positive - Ki-67: Extremely high (>50%)	- β-hCG: Strongly positive - p63: Negative or focal weak positive - Ki-67: Extremely high (>50%)	- β-hCG: Focal/weakly positive - p63: Diffuse nuclear positive (key feature) - Ki-67: Relatively low (10–25%; may reach 30% in hotspot areas, particularly in cases with scattered syncytiotrophoblast-like giant cells)
Histopathology	Biphasic cellular pattern (cytotrophoblasts + syncytiotrophoblasts)	Biphasic cellular pattern (identical to GC)	Monophasic cellular pattern (intermediate trophoblasts) with perinodular hyaline-like material deposition
Treatment	Chemotherapy-based (EMA-CO, PEB, etc.)	Surgery + adjuvant chemotherapy (lobectomy + lymph node dissection)	Primary complete surgical resection (uterine + pulmonary lesions); chemotherapy has limited efficacy
Response to Chemotherapy	Highly sensitive	Poor	Insensitive
Prognosis	Favorable	Poor	Relatively indolent; favorable after complete surgical resection

**Table 2:** Comparison of immunohistochemical tests for differential diagnosis of pulmonary ETT.

Marker	Pulmonary ETT	Pulmonary Squamous Cell Carcinoma	Pulmonary Adenocarcinoma	Malignant Melanoma	Lymphoma	Interpretation
p63	Diffuse, strong positive	Diffuse, strong positive	Negative/focal	Variable	Negative	Positive in both ETT and squamous cell carcinoma; requires combination with other markers
CK (AE1/AE3)	Diffuse positive	Positive	Positive	Positive	Negative	Epithelial marker; positive in ETT and carcinomas
p40	Positive	Positive	Negative	Negative	Negative	Similar to p63; ETT can mimic squamous cell carcinoma phenotype
β-hCG	Positive in multinucleated giant cells; negative in mononuclear cells	Negative (rare ectopic expression reported)	Negative	Negative	Negative	Key diagnostic marker for ETT; rare ectopic expression may occur in other tumors
hPL	Rarely positive in multinucleated cells	Negative	Negative	Negative	Negative	Trophoblastic differentiation marker
TTF-1	Negative	Negative	Positive	Negative	Negative	Key marker for distinguishing pulmonary adenocarcinoma
LCA	Negative	Negative	Negative	Negative	Positive	Key marker for distinguishing lymphoma
HMB-45	Negative	Negative	Negative	Positive	Negative	Key marker for distinguishing malignant melanoma
Melan-A	Negative	Negative	Negative	Positive	Negative	Key marker for distinguishing malignant melanoma
SOX-10	Negative	Negative	Negative	Positive	Negative	Key marker for distinguishing malignant melanoma
SALL4	May be positive (rare cells)	Negative	Negative	Positive	Negative	Germ cell/trophoblastic marker; may be positive in some melanomas
Ki-67	Pure ETT: <25% (may be up to 30% in hotspot areas); with coexisting choriocarcinoma: >70%	Variable	Variable	High	High	Proliferation index; aids in distinguishing pure ETT from choriocarcinoma

## Data Availability

The original contributions presented in this study are included in the article. Further inquiries can be directed to the corresponding author.

## Supplementary Materials

The following supporting information can be downloaded at: https://www.mdpi.com/article/10.3390/jcm15197578/s1. Table S1; Table S2. Clinical Timeline of Interventions and β-hCG Values.
