# Supplementary material for: Pulmonary Epithelioid Trophoblastic Tumor Without Demonstrable Uterine Involvement: A Case Report and Literature Review Highlighting Diagnostic Challenges and Multidisciplinary Management
Source: J Clin Med. 2026 Sep 29;15(19):7578. doi: 10.3390/jcm15197578 (PMC13665864; doi:10.3390/jcm15197578)
Supplement: Supplementary file 1 [file jcm-15-07578-s001.zip › jcm-4493792-supplementary.pdf]

Supplemental Table S1

Case	Age/yr	Symptoms	Preceding pregnancy	Interval from last pregnancy /mo	Pre-hCG（mIU/mL）	Surgical resection of pulmonary	Pre-chemotherapy	Post-chemotherapy	Follow-up, survival, mo
1	49	None	Hydatidiform mole	NA	NA	Thorascopic lower left lobectomy with mediastinal lymphadenectomy	-	-	3
2	38	Poor physical condition	Term pregnancy	12	80.1	Thorascopic right segmentectomy	6 cycles of EMA-CO	+	9
3	42	Hemoptysis and cough	Term pregnancy	NA	1300	VATS	-	Several courses after chemotherapy	24
4	31	None	Subclinical miscarriage	48	168.1	Thorascopic left upper lobe segmentectomy	-	3 cycles of EP-EMA	13
5	40	Vaginal bleeding	NA	NA	1100	Thorascopic lobe lobectomy with mediastinal lymph node dissection	-	4 cycles of cisplatin/etoposide	12
6	35	Abdominal pain, nausea, and vomiting	Term pregnancy	NA	NA	Thorascopic left lower lobectomy with mediastinal lymph node dissection	-	-	15
7	26	Delayed and relatively heavy menstruation	Suspected subclinical miscarriage	48	NA	Thorascopic right lower lobectomy with mediastinal lymph node dissection	-	6 cycles of EMA-CO	9
8	31	Irregular vaginal bleeding	Term pregnancy	96	700	Thorascopic right lower lobectomy and systematic mediastinal lymphadenectomy	-	-	12
9	28	None	Term pregnancy	48	50	Thorascopic wedge resection of the right lower lobe	-	3 cycles of TP/TE	47
10	29	vaginal bleeding	Term pregnancy	48	250	Unilateral ovariectomy,thorascopic left superior lobectomy	-	6 cycles of EP-EMA	12
11	32	Cessation of menstruation, vaginal bleeding, chest pain	Term pregnancy	24	75	Right upper wedge resection, Right lower segmentectomy and lynph node dissection	75mg MTX	-	12
12	38	None	Term pregnancy	42	400	Thorascopic right wedge resection and lobectomy	-	-	90

13	49	vaginal bleeding	miscarriage	12	2204	Right lower lobectomy with mediastinal lymph node dissection	3.5 cycles of EMA/EP	-	45
14	34	Irregular menses	Term pregnancy	24	426	Right upper lobe segmentectomy	4 cycles of MTX	3 cycles of EMA/EP	22
15	28	Vaginal bleeding	Molar pregnancy	17	2021	VAST in right lung	5 cycles of TP	2 cycles of EP-EMA+5 cycles of EP	NA
16	23	None	Abortion	11	227	VAST	-	3 cycles of EP/EMA	NA
17	32	Vaginal bleeding, cough	Term pregnancy	3	194	VAST	-	3 cycles of EP/EMA	NA
18	30	Vaginal bleeding	Molar pregnancy	14	144	VAST	-	3 cycles of EMA-CO	NA
19	47	None	Invasive mole	36	0.7	Thorascopic right upper lung lesion resection	-	-	24
20	32	None	Molar pregnancy	60	NA	Right upper lobectomy	-	-	36
21	42	Hemoptysis and cough	NA	NA	NA	Left upper lobectomy and lymph node dissection	-	+	24
22-27	30.5	NA	NA	18	Average213	Pulmonary lesion resection	NA	NA	30.5
28		NA	NA	NA	NA	Pulmonary lesion resection	+	-	/
29	31	Irregular vaginal bleeding	Abortion	12	304	Pulmonary lesion resection	+	-	/
30	39	Irregular vaginal bleeding	Term pregnancy	36	978	VAST in right lower lobectomy	1 cycle of MTX	3 cycles of EMA-CO	30

NA: not available, VAST: video assisted thoracoscopic surgery, TP: Paclitaxel, cisplatin，TE: docetaxel, epirubicin.

Supplemental Table S2. Clinical Timeline of Interventions and β-hCG Values.

Date	Event	β-hCG（mIU/mL）
2023-11-01	Last menstrual period	
2023-11-15	First detected β-hCG elevation **978**	978
2023-11-19	Follow-up β-hCG **1,100**	1100
2023-11-25	β-hCG (spontaneous decline) **433**	433
2023-12-13	β-hCG re-elevation **758**	758
2023-12-13	Laparoscopic bilateral salpingectomy + diagnostic curettage + IUD removal; non-diagnostic pathology	
2023-12-14	Postoperative β-hCG **859**	859

2023-12-20	β-hCG persistently elevated **993**	993
2023-12-21	β-hCG persistently elevated **1,094**	1094
2023-12-21	Chest CT: 5.7×40 mm right lower lobe mass (presumed primary lung cancer initially) ; The pelvic magnetic resonance imaging examination (MRI) did not reveal any significant abnormalities.	
2023-12-29	**VATS right lower lobectomy**; pathology: epithelioid trophoblastic tumor	
2024-01-05	Post-op day 7 β-hCG (near-normalization) **6.46**	6.46
2024-01-15	Post-op day 17 β-hCG normalized **3.13**	3.13
2024-01-18	Adjuvant chemotherapy cycle #1 (EMA-CO)	
2024-01-26	Post-op β-hCG (confirmed normal) **1.35**	1.35
2024-02-12	Adjuvant chemotherapy cycle #2 (EMA-CO)	3.59
2024-03-07	Adjuvant chemotherapy cycle #3 (EMA-CO)	2.89
2024–2027	Surveillance: undetectable β-hCG, no evidence of recurrence < 5	< 5
